# The role of SOCS proteins in the development of virus- induced hepatocellular carcinoma

**DOI:** 10.1186/s12985-021-01544-w

**Published:** 2021-04-13

**Authors:** Jinyan Xie, Mingshu Wang, Anchun Cheng, Renyong Jia, Dekang Zhu, Mafeng Liu, Shun Chen, XinXin Zhao, Qiao Yang, Ying Wu, Shaqiu Zhang, Qihui Luo, Yin Wang, Zhiwen Xu, Zhengli Chen, Ling Zhu, Yunya Liu, Yanling Yu, Ling Zhang, Xiaoyue Chen

**Affiliations:** 1 Institute of Preventive Veterinary Medicine, Sichuan Agricultural University, Wenjiang, Chengdu City, 611130 Sichuan People’s Republic of China; 2 Key Laboratory of Animal Disease and Human Health of Sichuan Province, Sichuan Agricultural University, Wenjiang, Chengdu City, 611130 Sichuan People’s Republic of China; 3 Avian Disease Research Center, College of Veterinary Medicine, Sichuan Agricultural University, Wenjiang, Chengdu City, 611130 Sichuan People’s Republic of China

**Keywords:** SOCS, Hepatocellular carcinoma, Cytokine, JAK-STAT signaling pathway, Hepatitis virus

## Abstract

**Background:**

Liver cancer has become one of the most common cancers and has a high mortality rate. Hepatocellular carcinoma is one of the most common liver cancers, and its occurrence and development process are associated with chronic hepatitis B virus (HBV) and hepatitis C virus (HCV) infections.

Main body

The serious consequences of chronic hepatitis virus infections are related to the viral invasion strategy. Furthermore, the viral escape mechanism has evolved during long-term struggles with the host. Studies have increasingly shown that suppressor of cytokine signaling (SOCS) proteins participate in the viral escape process. SOCS proteins play an important role in regulating cytokine signaling, particularly the Janus kinase-signal transducer and activator of transcription (JAK-STAT) signaling pathway. Cytokines stimulate the expression of SOCS proteins, in turn, SOCS proteins inhibit cytokine signaling by blocking the JAK-STAT signaling pathway, thereby achieving homeostasis. By utilizing SOCS proteins, chronic hepatitis virus infection may destroy the host’s antiviral responses to achieve persistent infection.

**Conclusions:**

This review provides recent knowledge regarding the role of SOCS proteins during chronic hepatitis virus infection and provides some new ideas for the future treatment of chronic hepatitis.

## Background

Although substantial progress has been achieved in modern medical standards, unknown and existing viruses still pose serious risks to human and animal lives. For example, the epidemics of hepatitis B virus (HBV) present an escalating trend of global infection risk which causes a large number of people to suffer from chronic HBV. More than 257 million people worldwide are chronically infected with HBV, even until now, the virus has not been completely eradicated [1]. Unfortunately, such infection can only be controlled. However, what is even worse, is that the outcomes of these infected with HBV are often being diagnosed as cirrhosis and hepatocellular carcinoma (HCC). HCV, another type of hepatitis virus that also poses serious threats to human health, its infection originates from an initial asymptomatic chronic infection which can lead to life-threatening cirrhosis or HCC [2]. The severity of these viral infections is due to the effective invasion strategy of these viruses, such as adhesion, invasion, and proliferation [3]. Furthermore, the virus has never stopped evolving escape mechanisms during the long-struggling with the host which spared them a better chance to evade the host's defense.

Some cytokines, such as Type I interferon, have direct or indirect antiviral effects on fighting virus infection. Cytokines constitute a class of signaling molecules secreted by cells that play critical regulatory roles in communication with surrounding cells [4]. Cytokines activate cell surface receptor complexes to regulate the activation, growth, differentiation, and apoptosis of cells [5]. These receptors are mainly divided into four categories. The first class of receptors primarily activates nuclear factor kappa-light-chain-enhancer of activated B cells (NF-κB) and mitogen-activated protein (MAP) kinases, which are mainly involved in the tumor necrosis factor α family, including the interleukin-1 family (IL-1β, IL-18, and IL-33) and the IL-17 family [6]. The second class of receptors primarily activates the Janus kinase-signal transducer and activator of transcription (JAK-STAT) signaling pathway, and most cytokines function by activating this signaling pathway [7]. The third class of receptors comprises the transforming growth factor β receptor, which mainly activates the Smad family of transcription factors [8]. The final class of receptors includes growth factor receptors, which primarily function through the Ras intracellular signal-regulated kinase pathway [9]. Cytokines activate intracellular signaling pathways by binding the corresponding receptors, but this process does not occur continuously because of a negative feedback regulation mechanism, which is important for homeostasis.

Cytokine-inducible SH2 (CIS) and suppressor of cytokine signaling (SOCS) proteins play important roles in regulating cytokine signaling, particularly the JAK-STAT signaling pathway. Numerous studies have found that cytokines stimulate the expression of SOCS proteins; however, SOCS proteins inhibit cytokine signaling by blocking the JAK-STAT signaling pathway, thereby achieving homeostasis [10]. In addition to cytokines, other stimuli, such as lipopolysaccharides, bacteria, viruses, and chemokines, induce the expression of SOCS proteins [11]. Additionally, empirical evidences have shown that a close relationship exists between the SOCS family and cancer [12–14]. According to the International Agency for Research on Cancer, liver cancer is one of the most common cancers with a high mortality rate. HCC is one of the most common liver cancers, and its occurrence and development processes are associated with cirrhosis induced by HBV and HCV infections [15]. In addition, an increasing number of studies indicate that SOCS proteins are involved in the development of HCC [16–18]. Up to this point, this review aims to summarize recent knowledge regarding the role of SOCS proteins during hepatitis virus infection, as well as contribute some new ideas for the future treatment of hepatitis.

## Immune regulation by SOCS

### CIS/SOCS family

**Fig. 1:**
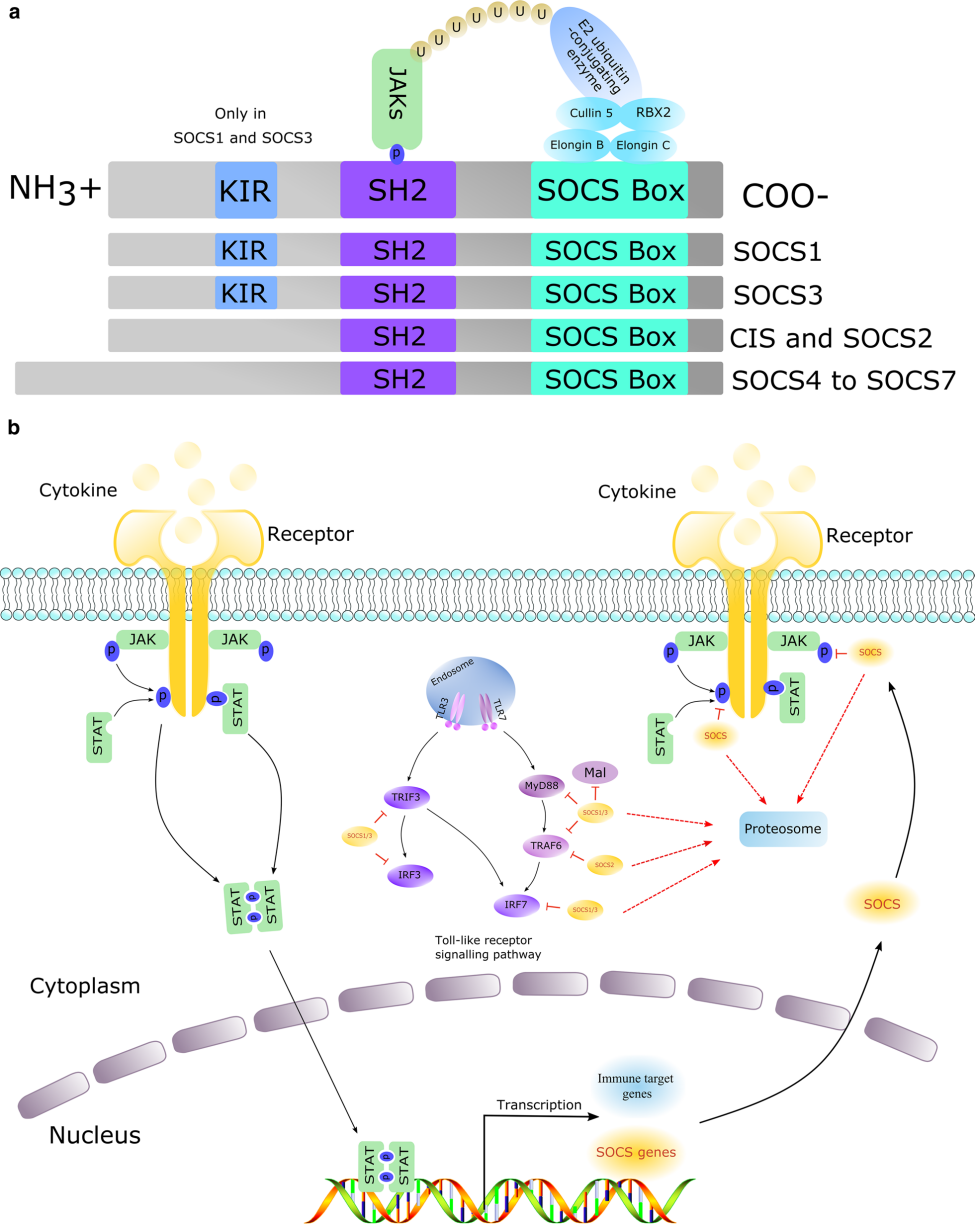
**a** SOCS protein structure. All SOCS proteins contain a central SH2 domain, an amino-terminal domain of variable length and a carboxy-terminal SOCS box. The SH2 domain recognizes and binds phosphorylated tyrosine residues on its specific substrate, such as JAK proteins. The SOCS box can interact with elongin B, elongin C, and cullin 5, utilizes the RING-finger-domain-only protein to recruit E2 ubiquitin-transferase, and ubiquitinates JAKs and other cytokine receptors, ultimately targeting them for proteasomal degradation. **b** Mechanism by which the SOCS proteins suppress the JAK-STAT pathway and TLR signaling pathway. Cytokines or interferons bind cellular membrane surface receptors, which activate and phosphorylate receptor-associated JAK proteins. Activated JAK proteins phosphorylate receptor cytoplasmic domains, which begin to recruit STATs, enabling their dimerization. Next, the dimerized complex enters the nucleus to initiate the transcription of different target genes, including the SOCS gene and immune effectors. SOCS proteins negatively regulate these pathways. In the JAK-STAT pathway, SOCS proteins compete with recruited STAT proteins for shared phospho-tyrosine residues or inhibit the activity of JAKs by the KIR domain of SOCS1 and SOCS3. Additionally, the SOCS box mediates the ubiquitination and degradation of bound receptor components. In the Toll-like receptor signaling pathway, SOCS1 and SOCS3 use the SH2 region to recognize and bind tyrosine-phosphorylated Mal, TNF receptor-associated factor 3/6 (TRAF3/6) and IRF7 [12, 17, 22]

The CIS/SOCS family includes CIS and SOCS proteins, can also further be subdivided into SOCS1, SOCS2, SOCS3, SOCS4, SOCS5, SOCS6, and SOCS7 [19, 20]. The proteins belonging to this family have similar structures: an SH2 domain is located in the central region, the amino terminus is of variable length and sequence, and the carboxy terminus is a module consisting of 40 amino acids, namely, the SOCS box [21]. These three-part structures have different functions; the SH2 domain recognizes and binds a cognate phospho-tyrosine residue, and different SOCS/CIS proteins use this domain to execute their regulatory functions. The amino-terminal region interacts with the substrate. However, the SOCS box interacts with elongin B, elongin C, and cullin 5, utilizes the RING-finger-domain-only protein (RBX2) to recruit E2 ubiquitin-transferase, and ubiquitinates Janus kinases (JAKs) and other cytokine receptors, ultimately targeting these proteins for proteasomal degradation (Fig. 1a) [17, 22, 23]. CIS is the first member identified in this family [24] and among the best-characterized member of the CIS/SOCS family. CIS was identified as a negative regulator of signal transducer and activator of transcription 5 (STAT5) in 1997 [25]. In a recent study, Cynthia Louis et al*.* found that CIS is a physiological inhibitor of GM-CSF signaling by restraining the inflammatory properties of myeloid cells [26].

SOCS proteins were originally shown to inhibit cytokine signaling through the JAK-STAT signaling pathway [27]. With more advanced research, SOCS proteins were found to play an important role in inflammation and the development and progression of cancers. The induction of the SOCS proteins is usually mediated by the JAK-STAT pathway. Following binding to cellular membrane surface receptors, cytokines and interferons activate and phosphorylate receptor-associated JAK proteins. Next, the activated JAK proteins phosphorylate the cytoplasmic domains of receptors. These phosphorylated receptors begin to recruit and activate STATs, enabling the dimerization of STATs. These dimerized complexes then enter the nucleus to initiate the transcription of different target genes, including the SOCS genes [17].

### Type I interferon signaling and the SOCS signaling pathway

Innate immunity is the first-line defense against invading pathogens and plays an important role in maintaining the host’s safety. The innate immune system includes the following three classical pattern-recognition responses (PRRs): Toll-like receptors (TLRs), retinoic acid-inducible gene I-like receptors (RLRs), and nucleotide oligomerization domain-like receptors (NLRs). Among these receptors, TLRs and RLRs play a pivotal role in the production of type I interferon and various cytokines, and NLRP3, which is the most widely studied NLRs, regulates the maturation of IL-1β and IL-18 via the activation of caspase-1.

After recognition, TLRs immediately recruit Toll/interleukin-1 receptor (TIR)-containing adaptors, such as MyD88 and TIR domain-containing adapters inducing IFN-β (TRIF), thus transmitting related signals to downstream molecules. Following the recognition of viral double-stranded RNA, the TLR3 signaling pathway is activated by the TRIF adaptor, and TNF receptor-associated factor 3 (TRAF3) is recruited in response. TRAF3 activates two related kinases, inducible I κB kinase (IKKi) and TANK-binding kinase 1 (TBK1), which, in turn, mediate the phosphorylation of interferon regulatory factor 3/7 (IRF-3/7). Finally, IRF-3/7 enters the nucleus and stimulates the production of type I interferon and cytokines. Type I interferon binds a heterodimeric transmembrane receptor, i.e., the IFNα receptor (IFNAR), which is composed of IFNAR1 and IFNAR2 subunits. Once IFNAR is bound by Type I interferon, the receptor-associated protein tyrosine kinases JAK1 and tyrosine kinase 2 (TYK2) are activated; the transcription factors STAT1 and STAT2 are then phosphorylated and form a dimer. Dimerized STAT1 and STAT2 assembles with IRF9 to form a trimolecular complex called IFN-stimulated gene factor 3 (ISGF3). Finally, ISGF3 translocates to the nucleus and binds its cognate DNA sequences called IFN-stimulated response elements (ISRE), thereby initiating the transcription of ISGs. [28–30]

Next, the SOCS proteins begin to express and play their roles in type I interferon signaling. CIS, SOCS1, SOCS2, and SOCS3 aim to execute their negative feedback regulation in JAK-STAT-mediated cytokine signaling, while SOCS4, SOCS5, SOCS6 and SOCS7 primarily focus on regulating the growth factor receptor signal. Among these proteins, SOCS1 and SOCS3 are the most widely studied [12]. SOCS1 and SOCS3 possess a unique kinase inhibitory region (KIR) at the amino terminus that inhibits the activity of JAK tyrosine kinase by acting as a pseudo substrate, meanwhile, the other SOCS proteins also directly or indirectly interact with JAKs or specific cytokine receptors using their respective structures, thereby degrading targeted proteins [31]. For example, SOCS1 and SOCS3 directly inhibit the activity of JAK tyrosine kinase through the KIR region, at the same time, SOCS proteins also block STAT by interacting with the phosphorylation region of the STAT receptor, thereby inhibiting cytokine signaling. Likewise, SOCS3 specifically inhibits STAT3 and STAT4 [17]. During the fine phase, the SOCS box region begins to recruit E2 ubiquitination transferase. Next, this complex ubiquitinylates the target proteins, marking them for proteasomal degradation. In addition, the SOCS proteins directly regulate the degradation of related proteins, such as E3 ubiquitination ligase [19].

In addition to regulating the JAK-STAT signaling pathway, SOCS proteins can also modulate the TLR signaling pathway. Similar to the negative feedback regulation of the JAK-STAT signaling pathway, SOCS proteins mainly bind key molecules in the Toll receptor signaling pathway and then use the SOCS box region to ubiquitinate and degrade the target molecule, thereby suppressing the signaling pathway. As SOCS1 and SOCS3 are amongst the most widely reported in the past studies. The findings have shown that SOCS1 and SOCS3 use the SH2 region to recognize and bind the myeloid differentiation factor 88 (MYD88)-adaptor-like (Mal) protein [32], TRAF3/6 [33] and a subunit of NF-κB p65 [34] that contains a phosphorylated tyrosine. In the same way, SOCS1 and SOCS3 bind and degrade IRF7, inhibiting the production of TLR7-mediated type I interferon [35]. SOCS3 regulates insulin signaling by binding insulin receptor substrate 1 and 2 (IRS1 and IRS2) proteins [36], and SOCS2 induces TRAF6 degradation (Fig. 1b) [37].

## Role of SOCS in hepatic viral infection

### SOCS signaling following the activation of innate immunity by viral infections

A complex balance system exists in the host that ensures that innate and adaptive immune responses are properly activated and terminated via the regulation of positive and negative signaling to clear invading viruses and regulate uncontrolled inflammation. However, persistent pathogen infection may disrupt this balanced mechanism to achieve long-term stable replication and proliferation in the host. Host cells recognize the components of a virus through PRRs and activate innate immune responses, while adaptive immunity plays an important role in preventing the reinvasion of pathogens. Once a virus invades the host, PRRs quickly recognize the virus, activate complex intracellular signaling pathways and target inflammatory and antiviral responses, ultimately removing the invading virus rapidly. TLRs not only activate the host's innate immunity but also promote specific adaptive immunity by acting on antigen presenting cells. Among these processes, cytokine production is inseparable from the key signaling pathway, i.e., the JAK-STAT signaling pathway. The JAK-STAT signaling pathway regulates more than 50 downstream cytokines and growth factors as a central communication node in the immune system. This signaling pathway mainly comprises JAK and STAT proteins; JAKs can be subdivided into JAK1, JAK2, JAK3 and TYK2, while STATs can be subdivided into STAT1, STAT2, STAT3, STAT4, STAT5A, STAT5B and STAT6 [38]. As a negative feedback regulator of the JAK-STAT signaling pathway, SOCS proteins play an important role in viral infection. SOCS proteins regulate the viral-induced cytokine levels by initiating the JAK-STAT signaling cascade and interfere with cellular signaling by regulating the degradation of signaling proteins. These signaling proteins regulate immune cells to produce large amounts of pro- and anti-inflammatory cytokines, which further determine the host's sensitivity to viral infections and host outcomes after viral infection [39].

### HCV and SOCS proteins

HCV was the first virus used to explore the function of SOCS proteins, which were revealed to inhibit type I interferon signaling. Once HCV stably proliferates in the liver, the virus can compete with the antiviral response produced by the host and interact with various immune evasion mechanisms to damage the host's antiviral response, such as the type I interferon response. A recent study revealed that HCV increased SOCS1 expression as the viral load increased [40]. HCV infection upregulates SOCS proteins via different mechanisms depending on different HCV proteins. In particular, The HCV protein p7 induces SOCS3 via STAT3 and extracellular signal-regulated kinase (ERK)-mediated pathways [41]. On the other hand, SOCS7 expression induced by the HCV core protein genotype 3a is independent of STAT3 and may be regulated by peroxisome proliferator-activated receptor gamma (PPAR-γ) activity [42]. However, the mechanism by which the HCV core protein induces STAT3 or PPAR-γ still remains uncertain. Therefore, such subject is worthy of further investigation. The related research outcomes can provide meaningful implications for the future treatment of HCV infection.

Due to the fact that HCV infection mainly evades host innate immunity by regulating SOCS proteins. Overexpressed SOCS1 protein also inhibits the antiviral effect of type I interferon [43] and type III interferon (IFN-λ) signaling [44, 45], which inhibits the IFNα/λ-induced activation of the JAK/STAT signaling pathway by downregulating the phosphorylation of STAT1 and STAT3. SOCS1 and SOCS3 proteins inhibit IFN-α induced expression of the anti-viral proteins 2′,5′-OAS and MxA in Hep-G2 cells [43]. Hepatic SOCS3 expression is increased in patients infected with HCV genotype 1, which may be a mechanism by which SOCS3 reduces the biological response to IFN-α [46]. In addition to inhibiting innate immunity signaling, different HCV genotypes have different effects on adaptive immunity by regulating SOCS proteins. The HCV core protein differentially regulates T- and B-cell signaling by exploiting programmed death-1 (PD-1) and SOCS1 functions [47, 48]. A previous report provided evidence that the HCV core protein inhibits T-cell responses by interacting with gC1qR, inducing SOCS1/3 and suppressing STAT1/3 [49, 50]. However, B cells are abnormally activated during chronic HCV infection, characteristics associated with TNF- and Apo-L-related leucocyte-expressed ligand-1 (TALL-1) overexpression and SOCS-1 suppression [51]. Another study revealed that patients with HCV genotype 3 sustained virological responses (SVR) have significantly higher expression of SOCS or lower expression of MHC class-II genes, which is associated with a diminished capacity to present antigens and a functionally defective state of dendritic cells [52]. However, a recent study revealed that the transcript levels of SOCS1 in peripheral blood mononuclear cells do not significantly differ between 100 HCV genotype 1 patients with SVR and non-SVR [53]. Another study revealed that a specific strain of HCV, i.e., genotype 1b, fails to activate the transcription of SOCS1, SOCS3, and SOCS7. Furthermore, amino acids 49 and 131 of the HCV core-encoding sequence mediate these transactivating effects [54].

Patients with chronic HCV infection often have multiple associated diseases, several of which have found to be linked to SOCS protein expression. Empirical epidemiological studies show strong evidences in which HCV infection is linked to type 2 diabetes, which is thought to originate from the downregulation of IRS1 and IRS2 by upregulating SOCS3 during HCV infection [55, 56]. Furthermore, the HCV core protein of genotype 3a promotes IRS1 (but not IRS2) degradation by upregulating SOCS7 in a human hepatoma cell line (Huh-7) [57]. Another report revealed that the HCV core protein in both transgenic mice or hepatoma cells downregulates IRS1 and IRS2 by upregulating SOCS3 [56]. These data suggest that HCV core-induced SOCS3 and SOCS7 promote the proteasomal degradation of IRS1 and IRS2 through ubiquitination, thereby contributing to HCV-induced insulin resistance. Furthermore, HCV suppresses proinflammatory TNF-α responses by inducing SOCS3 [58].

Recently, numerous reports have shown that miRNAs play an important role in hepatitis virus infections. In these reports, miR146a is upregulated in monocytes from HCV-infected patients [18], and miRNA221 is upregulated in serum from HCV chronic hepatitis patients and Huh7.5.1 cells infected with HCV [59]. Furthermore, miRNA164a increases IL-23, IL-10, and TGF-β1 expression by inhibiting SOCS1 and inducing STAT3 [18]. In addition, miRNA221 enhances the action of interferon against HCV by targeting SOCS1 and SOCS3 [59]. miRNA122 also regulates type I interferon by blocking SOCS1 [60]. These phenotypes demonstrate that the host reacts to HCV via certain miRNAs targeting SOCS proteins against HCV. However, HCV employs multiple strategies to evade host immune responses by hijacking SOCS proteins (Table 1).

**Table 1:** Hepatitis viruses have developed multiple strategies to disrupt the immune response by hijacking the SOCS system

Virus	SOCS protein	In vivo or In vitro	Viral proteins	Mechanism	References
HCV	SOCS1/3	Hep-G2 cell	HCV core protein	Inhibit IFN-α induced expression of 2′,5′-OAS and MxA	[40]
	SOCS3	patients	HCV genotype 1	Reduce the biological response to IFN-α	[43]
	SOCS1	PBMC	HCV core protein	Dysregulate T- and B-cell signalling	[44–47]
	SOCS	Myeloid DCs	HCV genotype 3	Diminish capacity to present antigen	[49]
	SOCS3/7	Huh-7	HCV genotype 3	Downregulation of IRS1 and IRS2	[52–54]
HBV	SOCS3	Huh-7 and liver specimens from HBV-infected patients	adenoviral AdHBV (genotype A)	Dysregulate STAT/SOCS-signalling	[58]
	SOCS1	HepG2.2.15 cells and PBMC	HBV antigen, HBeAg	Promote inflammatory cytokine production	[60]
	SOCS1	plasmacytoid dendritic cells	HBsAg	Inhibition of the IFN-α production	[61]
	SOCS1/3	HBV transgenic mice	CTP-HBcAg18-27-tapasin	enhance T cell immune responses	[62–64]

Viral proteins disrupt the host’s immune response by hijacking different SOCS proteins in vivo or in vitro.

### HBV and SOCS proteins

In another hepatitis virus study, HBV infection increased SOCS3 expression in Huh7 cells, resulting in sustained STAT3 activation [61]. Additionally, the woodchuck model has been used to study HBV, and woodchuck hepatitis virus (WHV) elevates the mRNA levels of SOCS3 in the liver during chronic WHV infection [62]. Uncontrolled SOCS and STAT signaling result in more severe inflammation in the liver, which may be related to persistent HBV infections [61]. A recent report showed that an HBV antigen, i.e., HBeAg, augments miR-155 expression in macrophages, and increased miR-155 levels promote HBeAg-induced inflammatory cytokine production by suppressing SOCS1 expression in HepG2.2.15 cells [63]. However, another HBV antigen, i.e., HBsAg, significantly induces the expression of SOCS1, which may be a key factor in the HBsAg-regulated inhibition of TLR9-mediated signaling; furthermore, HBsAg binds BDCA2 receptors on the plasma membrane of pDCs, resulting in the inhibition of IFN-α production [64]. Additionally, Y Tang et al. reported that the fusion protein of cytoplasmic transduction peptide (CTP) and HBV core antigen 18-27 (HBcAg 18-27)-Tapasin efficiently enhances T-cell immune responses in vitro. Mechanically, they found that CTP-HBcAg18-27-Tapasin enhances the Th1/Th2 cytokine ratio and antiviral immunity by suppressing SOCS1 and SOCS3 in HBV transgenic mice [65–67]. These data indicate that different HBV antigens play different roles in regulating SOCS proteins. However, the mechanism by which HBV-antigens induce SOCS proteins, particularly SOCS1, thereby inhibiting the anti-viral response, must be explored more extensively. For example, how does HBsAg induce SOCS1 expression? HBsAg may rely on the same pathway as the HCV core protein, inducing SOCS proteins via STAT3 and ERK-mediated pathways. Notably, HBsAg and other HBV antigens more likely interact with certain proteins to induce SOCS proteins.

HBV inhibits the level of endogenous interferon and induces SOCS1 and SOCS3 expression; however, following interferon treatment, HBV replication is inhibited, and the expression levels of SOCS1 and SOCS3 begin to decrease, indicating that HBV may achieve sustained infection in vivo by upregulating the expression of the SOCS proteins [68]. SOCS1 expression is negatively correlated with the prognoses of patients with acute-on-chronic hepatitis B liver failure (ACHBLF); meanwhile, patients without SOCS1 methylation display a favorable response to glucocorticoid treatment [69]. Furthermore, HBV-X mutants enhance STAT3 activation, inhibit STAT1 expression and silence SOCS1 and SOCS3 expression, likely because of the atypical nuclear/perinuclear localization of the HBV-X mutants [70]. Furthermore, SOCS1 polymorphisms may affect the susceptibility and outcome of HBV infection [71], and CIS polymorphisms are associated with persistent HBV infection [72]. Most patients with chronic HBV infection develop HCC. Increasing evidence indicates that the X protein of HBV is closely related to HCC. In addition, the X gene of HBV downregulates the SOCS1 expression but increases SOCS-1 gene promoter CpG island methylation, leading to oncogenes activation and HCC development [73]. X. ZHANG et al*.* also found that the gene loss and epigenetic silencing of SOCS1 are strongly associated with HBV-related HCC [14]. However, the hypermethylation of CpG islands of SOCS-1 is not closely related to HBV-induced HCC [13]. Furthermore, the expression of SOCS family proteins is remarkably suppressed in the livers from HBV X protein transgenic mice relative to that in non-transgenic mice from the early to late stages of partial hepatectomy (PH) compared with non-PH mice [74]. The relationship between HCC and SOCS proteins remains largely unknown. In summary, innate and adaptive immunity may be disrupted during HBV infection by enhanced levels of SOCS proteins, leading to persistent HBV infection (Table 1).

### Other hepatitis viruses and SOCS proteins

Only a few studies have reported the interactions between SOCS proteins and hepatitis A virus (HAV). However, in various animal models of HAV, SOCS proteins are involved in HAV infection. For example, previous reports have shown that ducks can be used as a small animal model for studying HAV, and duck hepatitis A virus (DHAV) can induce acute and chronic hepatitis in ducks [75]. During DHAV-1 infection, the liver expresses a large amount of SOCS1 and SOCS3, which may be involved in viral adaption [76]. Furthermore, DHAV-1 inhibits type I interferon signaling to assist viral adaption by increasing SOCS3 expression [77].

### SOCS proteins as therapeutic targets to treat chronic hepatitis

Considering the relationships between chronic hepatitis and SOCS proteins, small-molecule antagonists of SOCS signaling and SOCS protein silencing could be viable strategies for improving the treatment of chronic hepatitis as well as effectively mitigating hepatitis virus infection. Waiboci et al*.* found that the KIR region of SOCS-1 binds directly to the phosphorylation site of JAK2 and they synthesized a peptide of pJAK2 (1001–1013, LPQDKEYYKVKEP) as an antagonist of SOCS1 [78]. This antagonist enhances innate and adaptive immune response by targeting the KIR of SOCS1 [79]. HBV and HCV evade host immune responses by hijacking SOCS proteins. Therefore, the SOCS1 antagonist may be valuable to treat chronic hepatitis. Furthermore, IL-7 downregulates SOCS3, resulting in amplified cytokine production, increases T-cell effector function and numbers, and attenuates viral persistent infection [80]. HBV infection disrupts innate and adaptive immunity by enhancing the levels of SOCS proteins, causing persistent HBV infection. These attributes of SOCS protein inhibition by an antagonist (pJAK2 peptide) or natural cytokines (IL-7) have profound implications for the therapeutic in the treatment of chronic hepatitis.

## Conclusions

In recent years, we have enhanced our understanding of the structure and function of SOCS proteins by conducting numerous in-depth researches. By functioning as negative feedback regulators, SOCS proteins are involved in the participation of the life cycle of the host, especially during the process of hepatitis virus infection. Studying the role of SOCS proteins in hepatitis virus-induced hepatocellular carcinoma provides some new ideas for improving the future treatment of hepatitis.

## Data Availability

Not applicable.

## Abbreviations

HCC: Hepatocellular carcinoma
HBV: Hepatitis B virus
HCV: Hepatitis C virus
NF-κB: Nuclear factor kappa-light-chain-enhancer of activated B cells
MAP: Mitogen-activated protein
SOCS: Suppressor of cytokine signaling
JAK-STAT: Janus kinase-signal transducer and activator of transcription
CIS: Cytokine-inducible SH2
RBX2: RING-finger-domain-only protein
PRRs: Pattern-recognition responses
TLR: Toll-like receptor
RLRs: Retinoic acid-inducible gene I-like receptors
NLRs: Nucleotide oligomerization domain-like receptors
KIR: Kinase inhibitory region
TRIF: TIR domain-containing adapters inducing IFN-β
TRAF: TNF receptor-associated factor
IRF: Interferon regulatory factor
IKKi: Inducible I κB kinase
TBK1: TANK-binding kinase 1
IFNAR: IFNα receptor
ISGF3: IFN-stimulated gene factor 3
ISRE: IFN-stimulated response elements
IRS: Insulin receptor substrate
IFN-λ: Type III interferon
PD-1: Programmed death-1
TALL-1: Apo-L-related leucocyte-expressed ligand-1
SVR: Sustained virological responses
HAV: Hepatitis A virus
DHAV: Duck hepatitis A virus
WHV: Woodchuck hepatitis virus
